# Practical recommendations for infectious prophylaxis and vaccination in multiple myeloma patients

**DOI:** 10.3389/fmed.2026.1847793

**Published:** 2026-06-30

**Authors:** Rui Bergantim, Adriana Roque, Ana Jorge, José Guilherme Freitas, Mariana Trigo Miranda, Marta Nunes, Alina Ionita, Paulo Varanda Bernardo, Joana Vieira, Eliana Vale Aguiar, Patrícia Ferraz, André Silva-Pinto, Ana Bela Sarmento-Ribeiro, Catarina Geraldes, Cristina João

**Affiliations:** 1 Clinical Hematology Department, Unidade Local de Saúde São João, Porto, Portugal; 2 Cancer Drug Resistance Group, Institute of Molecular Pathology and Immunology (IPATIMUP), Universidade do Porto, Porto, Portugal; 3 i3S - Institute for Research and Innovation in Health, Universidade do Porto, Porto, Portugal; 4 Hematological and Oncological Diseases Unit, Medicine Department, Faculty of Medicine of the University of Porto, Porto, Portugal; 5 Clinical Hematology Department, Unidade Local de Saúde de Coimbra, Coimbra, Portugal; 6 Physiology Institute, Faculdade de Medicina, Universidade de Coimbra, Coimbra, Portugal; 7 Clinical Hematology Department, Unidade Local de Saúde Lisboa Ocidental, Lisbon, Portugal; 8 Clinical Hematology Department, Unidade Local de Saúde de Braga, Braga, Portugal; 9 Clinical Hematology Department, Hospital De São Teotónio, Unidade Local de Saúde Viseu Dão-Lafões, Viseu, Portugal; 10 Clinical Hematology Department, Unidade Local de Saúde Gaia e Espinho, Vila Nova de Gaia, Portugal; 11 Hematology Department, Instituto Português de Oncologia Francisco Gentil de Lisboa, Lisbon, Portugal; 12 Hematology and Bone Marrow Transplantation Department, Unidade Local de Saúde de Santa Maria, Lisbon, Portugal; 13 Hematology Department, Unidade Local de Saúde de Trás-Os-Montes e Alto Douro, Vila Real, Portugal; 14 Infectious Diseases Department, Unidade Local de Saúde São João, Porto, Portugal; 15 Infectious Diseases Unit, Medicine Department, Faculty of Medicine of the University of Porto, Porto, Portugal; 16 Laboratory of Oncobiology and Hematology (LOH), University Clinics of Hematology and Oncology, Faculty of Medicine (FMUC), University of Coimbra, Coimbra, Portugal; 17 Coimbra Institute for Clinical and Biomedical Research (iCBR)-Group of Environmental Genetics of Oncobiology (CIMAGO), FMUC, University of Coimbra, Coimbra, Portugal; 18 Center for Innovative Biomedicine and Biotechnology (CIBB), Coimbra, Portugal; 19 Hematology Service, Centro Hospitalar Universitário de Coimbra, Unidade Local de Saúde de Coimbra, Coimbra, Portugal; 20 Hemato-Oncology Unit, Fundação Champalimaud, Lisbon, Portugal; 21 NOVA Medical School, NOVA University of Lisbon, Lisbon, Portugal

**Keywords:** CAR-T cell therapy, immunoglobulin replacement, multiple myeloma, prophylaxis, secondary immunodeficiency

## Abstract

Infections remain a major cause of morbidity and mortality in multiple myeloma (MM), driven by the interplay between disease-induced immune dysfunction and treatment-related immunosuppression. The introduction of proteasome inhibitors, immunomodulatory drugs, monoclonal antibodies, bispecific antibodies, and chimeric antigen receptor T (CAR-T) cell therapy has markedly improved survival while simultaneously reshaping the infectious risk landscape. Different treatment strategies compromise immunity in distinct mechanisms. Conventional chemotherapy and some targeted agents primarily increase the risk of bacterial infections through neutropenia. Modern immunotherapies, however, produce profound and prolonged hypogammaglobulinemia alongside cellular immune deficits, predisposing patients to viral reactivation and opportunistic infections. BCMA-targeting therapies (particularly bispecific antibodies and CAR-T cells) cause sustained plasma cell depletion and durable humoral immune impairment, resulting in an infection pattern that can persist well beyond treatment completion. Effective prevention requires a risk-stratified approach accounting for disease stage, treatment regimen, cumulative immunosuppression, and individual patient characteristics. This review synthesizes current evidence and provides practical, treatment-specific recommendations spanning infection risk assessment, pre-treatment screening, antimicrobial and antifungal prophylaxis, antiviral strategies, immunoglobulin replacement, granulocyte colony-stimulating factor use, and vaccination. Emphasis is placed on bispecific antibodies and CAR-T cell therapies, where infectious risk is greatest and prophylactic strategies are evolving most rapidly. Immunoglobulin replacement is highlighted as an increasingly relevant supportive strategy, with recent observational studies linking its use to marked reductions in serious infections and, among recipients of anti-BCMA bispecific antibodies, improved survival. This review provides clinicians with a practical framework for individualized infection prevention in the evolving therapeutic landscape of MM.

## Introduction

Over the past two decades, the treatment landscape of multiple myeloma (MM) has been transformed by the introduction of novel drug classes and therapeutic strategies, resulting in significant improvements in progression-free survival (PFS) and overall survival (OS). However, as patients live longer and receive successive lines of therapy, the cumulative impact on immune function has become an increasingly critical consideration, particularly regarding infection risk.

**Figure 1:**
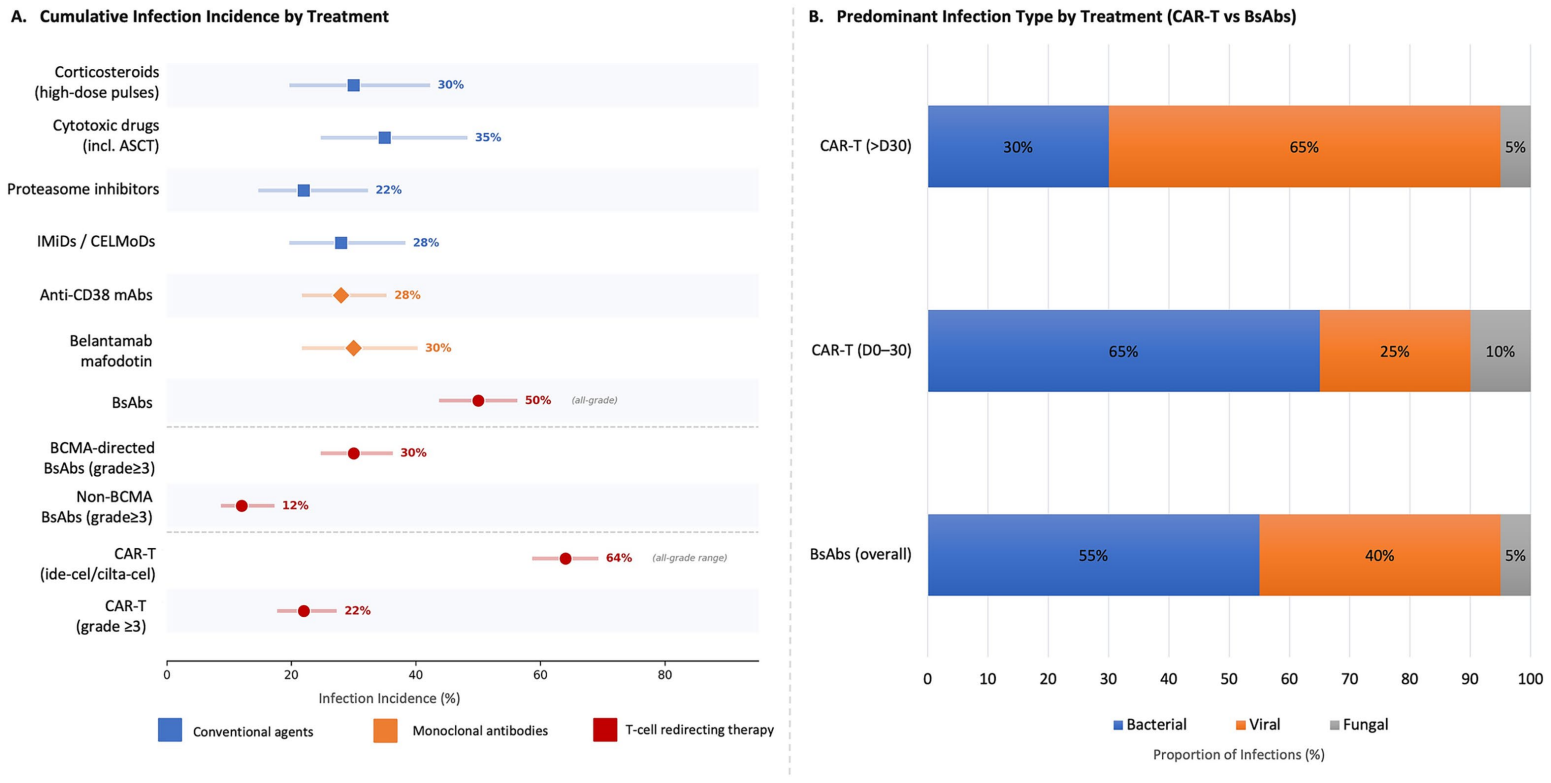
Infection risk by treatment class in multiple myeloma. **(A)** Cumulative infection incidence (%) by treatment class, derived from published clinical trials and meta-analyses. Horizontal bars represent approximate reported ranges (95% Cl or interquartile range where available). **(B)** Proportional distribution of infection type (bacterial, viral, and fungal) by treatment class (CAR-T vs. BsAbs), based on pooled data from prospective and retrospective studies. Infections are categorized by predominant pathogen class reported in the respective studies. Values are approximate and reflect broad treatment-class patterns; direct cross-trial comparisons are not intended. The references for this figure available in Supplementary Table 1. ASCT, autologous stem cell transplantation; BsAb, bispecific antibody; CAR-T, chimeric antigen receptor T-cell; CELMoD, cereblon E3 ligase modulator; IMiD, immunomodulatory drug; mAb, monoclonal antibody.

The risk of infection persists throughout the course of MM, but its magnitude, timing, and microbiological spectrum vary with disease status and treatment exposure. This evolving, treatment-specific infectious landscape is summarized in Figure 1. A population-based study investigated infection incidence in 8,672 symptomatic MM patients diagnosed between 2008 and 2021 and 34,561 individually matched controls. Overall, MM patients had a 5-fold higher risk of developing a clinically significant infection compared with controls (HR = 5.30; 95% CI: 5.14–5.47). Bacterial infections drove much of this burden, with a 5-fold excess risk (HR = 4.88; 95% CI: 4.70–5.07), while viral and fungal infections conferred an approximately 7-fold increased risk. The infection burden was most pronounced in the first year after diagnosis, when overall risk rose to 7-fold compared with controls (HR = 6.95; 95% CI: 6.61–7.30) and remained elevated throughout the 5-year follow-up (1). These population-level findings are supported by clinical trial data: A systematic review and meta-analysis of 31 randomized controlled trials measured the monthly risk of grade III or higher infections across all treatment phases, showing a consistent rate of approximately 1.7% per month in the frontline setting and 1.5% per month in the relapsed/refractory setting (2). A recent study of 148 MM patients reported 345 infections, about half of which were viral, over roughly a year (3). Another study of 479 patients found that 65% had at least one infection, most of which were bacterial (4). With successive lines of therapy, this risk does not plateau: T-cell redirecting therapies, such as bispecific antibodies (BsAbs) and chimeric antigen receptor T (CAR-T) cell therapies, are now associated with cumulative infection rates exceeding 50–60%, driven by the profound B-cell aplasia, hypogammaglobulinemia, and sustained T-cell lymphopenia that characterize their mechanism of action. A systematic review and meta-analysis of 16 clinical trials comprising 1,666 patients treated with BsAbs found a pooled all-grade infection prevalence of 56%, with grade ≥3 infections accounting for 24% of cases (5). In a single-center retrospective study, the overall incidence of infections was 76% with CAR-T and exceeded 80% across BsAbs cohorts, although most events were low grade. When comparing the two T-cell redirecting modalities directly, severe infections were more frequent with BsAbs (40%) than with CAR-T (26%), including fatal infections in 7% of BsAbs recipients (6). Notably, the spectrum of infection has shifted, with an increase in severe opportunistic and viral reactivation syndromes that require vigilance (7, 8). Infections significantly affect prognosis, treatment, and mortality, causing delays or discontinuations, and account for up to 27–32% of early deaths and 20% in the first year (1, 2).

In this context, preventive strategies to reduce and anticipate infectious complications are a crucial component of supportive care in MM. This narrative reviews the key patient-, disease-, and treatment-related risk factors for infection and provides evidence-based, practical recommendations for antimicrobial prophylaxis, vaccination, granulocyte colony-stimulating factor (G-CSF) use, immunoglobulin replacement, and infection screening, stratified by disease stage and treatment regimen.

## Risk factors

The risk of infection in an immunosuppressed patient always results from an intersection of factors related to the patient (such as comorbidities, previous infections, and performance status), the disease (its nature and stage), and the treatment. The main disease, patient, and treatment-related risk factors are summarized in Table 1.

**Table 1:** Risk factors for infection in patients with multiple myeloma.

Risk factors	Mechanism increasing infection risk
Disease-related	
Immunoparesis and hypogammaglobulinemia	Impaired humoral immunity and reduced antibody production
T-cell dysfunction	Suppressed lymphocyte activation and increased checkpoint expression
High tumor burden (ISS stage II–III, elevated LDH)	Suppression of normal hematopoiesis and immune dysfunction
Bone marrow infiltration	Reduced immune cell production
Relapsed/refractory disease	Cumulative immune exhaustion; profound B-cell aplasia; T-cell dysfunction; limited treatment options
Patient-related	
Advanced age	Immune senescence, reduced naive T-cell output and reduced vaccine response, and organ dysfunction
Poor performance status and frailty	Reduced physiological and immunological reserve with limited treatment tolerability
Renal or respiratory impairment	Increased susceptibility to severe infections
Prior or recurrent infections	Indicator of impaired immune competence and predispose to repeat episodes
Incomplete vaccination	Reduced protection against preventable infections
Treatment-related	
*Corticosteroids*	
High cumulative corticosteroid exposure	Suppression of lymphocyte activation and B/T-cell production, promotion of lymphocyte apoptosis and impaired phagocyte function
*T-cell redirecting therapies*	
Bispecific antibodies –BCMA—targeting (teclistamab and elranatamab)	Profound B-cell aplasia; sustained hypogammaglobulinemia (>75%); T-cell exhaustion; CRS/ICANS and their treatment
Bispecific antibodies non-BCMA—(talquetamab, cevostamab)	T-cell exhaustion; CRS/ICANS; less profound hypogammaglobulinemia than BCMA-targeting
CAR-T cell therapy	Lymphopenia and neutropenia. Hypogammaglobulinemia. CRS/ICANS
*Transplant and intensive chemotherapy*	
Myeloablative conditioning (pre-ASCT)	Severe neutropenia. Mucositis disrupts gastrointestinal barrier. Prolonged immune reconstitution required
Intensive chemotherapy (PACE-like regimens)	Prolonged severe neutropenia; mucositis; cumulative immunosuppression
Lymphodepleting regimens (fludarabine/cyclophosphamide pre-CAR-T)	Profound cellular immunosuppression; prolonged lymphopenia
*Monoclonal antibodies and ADCs*	
Anti-CD38 mAbs	CD38 expression on normal immune effector cells leads to off-tumor depletion, reduction in polyclonal Ig, and impaired NK cell surveillance
ADCs (belantamab mafodotin)	Depletion of BCMA-expressing plasma cells
*IMiDs/CELMoDs*	
IMiDs	Impaired neutrophil maturation despite myeloid colony expansion. Hematologic toxicity
CELMoDs (iberdomide and mezigdomide)	Higher-grade neutropenia (up to 50–60%); CMV reactivation; invasive aspergillosis reported
*Proteasome inhibitors*	
Proteasome inhibitors	NF-κB inhibition with transient CD4+ lymphocyte depletion and T-cell apoptosis. Hematologic toxicity
*Other agents*	
Selinexor; Venetoclax	Neutropenia
*Cumulative factors*	
Multiple prior treatment lines	Progressive immune exhaustion

ADC, antibody–drug conjugate; ASCT, autologous stem cell transplantation; BCMA, B-cell maturation antigen; CAR-T cell, chimeric antigen receptor T-cell; CD4+, cluster of differentiation 4; CD38, cluster of differentiation 38; CELMoD, cereblon E3 ligase modulator; CRS, cytokine release syndrome; ICANS, immune effector cell-associated neurotoxicity syndrome; Ig, immunoglobulin; IMiD, immunomodulatory drug; ISS, International Staging System; LDH, lactate dehydrogenase; mAb, monoclonal antibody; NF-κB, nuclear factor kappa B; NK, natural killer; PACE, cisplatin doxorubicin cyclophosphamide etoposide; T-cell, T lymphocyte.

### Related to multiple myeloma and immunoparesis

MM causes global immunoparesis, impairing immune components and leading to progressive dysfunction, impaired anti-tumor responses, angiogenesis, and drug resistance, especially in relapsed cases treated with immunotherapy (8, 9).

Immunologically, there is an increase in the expression of inhibitory molecules, such as programmed death-ligand 1 (PD-L1), not only in clonal plasma cells but also in macrophages and other antigen-presenting cells, and an increase in the expression of checkpoints in lymphocytes and natural killer (NK) cells in the MM niche (10). Suppressor macrophages and myeloid suppressor cells infiltrate the tumor microenvironment, and dendritic cells are functionally suppressed due to reduced levels of stimulatory molecules (11). Considering T lymphocyte populations, there is increased expression of negative co-stimulation molecules, such as T-cell immunoreceptor with Ig and ITIM domains (TIGIT) and PD1, an increased number of regulatory B and T lymphocytes in the medullary niche, and an alteration of the normal Th1/Th2 functional ratio (11). In the B lymphocyte compartment, neoplastic cells also impair normal B lymphocyte differentiation, thereby inhibiting polyclonal B lymphocyte responses and causing hypogammaglobulinemia (12).

The advanced International Staging System (ISS) stage (especially stage III) or a high disease burden, characterized by elevated serum lactic dehydrogenase (LDH), is associated with a notably higher risk of infections (13). This association likely reflects the deeper immunoparesis and more profound suppression of normal hematopoiesis that accompany high tumor burden, compounding baseline infection vulnerability (2).

Recent high-dimensional immune profiling studies indicate that infection susceptibility in MM reflects a quantifiable state of immune competence that can be captured through composite immune biomarkers. Specific alterations, including reduced CD27^+^ B cells, decreased CD27^−^ NK cells, and increased CD27^−^/CD27^+^ T-cell ratios, have been associated with impaired immune surveillance and increased infection risk. These parameters can be integrated into an immune risk score that stratifies patients by infection susceptibility, with individuals harboring ≥2 immune risk factors demonstrating substantially higher infection incidence than those with fewer abnormalities (approximately 60% vs. 35%), independent of disease stage or therapy exposure (14). Importantly, immune signatures correlate between bone marrow and peripheral blood, enabling minimally invasive monitoring via circulating immune cell profiling (15). While promising, high-dimensional immune profiling remains largely unavailable in routine clinical practice, limiting the immediate applicability of immune risk scoring. In MM, infection risk remains multifactorial, so these tools should complement, rather than replace, comprehensive clinical assessment.

### Patient-related factors

Several patient-specific characteristics also influence the risk of infection. Poor performance status indicates a likelihood of early and severe infections. Furthermore, comorbidities such as renal and respiratory impairment can increase the risk of infections (16). Older age is associated with immune senescence and a higher risk of infection, as well as reduced antibody responses to vaccines (13). Low vaccination rates for influenza and *Streptococcus pneumoniae* are associated with higher proportions of infection-related deaths (17). Male sex has been identified as a risk factor for pneumonia and sepsis in MM, although findings across studies are inconsistent, with some reporting a significantly lower infection risk in female patients and others finding no meaningful sex-based difference (9, 13, 16). A history of frequent or prior infections, including those during BsAbs therapy, is a consistent marker of subsequent infectious risk (18).

### Treatment-related factors

*Corticosteroids*: Corticosteroids play a crucial role in both upfront and relapsed/refractory (RRMM) treatment, but, depending on dose and duration, they exert a complex immunosuppressive effect, suppressing lymphocyte activation, promoting apoptosis, and inhibiting B- and T-cell production (19–21). We should take two variables into account: the dose and the duration of the corticotherapy. High cumulative prednisolone-equivalent doses exceeding 1,600 mg were associated with an increased risk of bacterial infection and those exceeding 3,200 mg with a high risk of viral infection (1, 22). In fact, the high-dose dexamethasone pulses commonly used in MM regimens, such as 40 mg for 4 consecutive days or weekly, or a median dosage of 13 mg prednisolone/day, confer particular risk of *Pneumocystis jirovecii* pneumonia (PJP) and VZV reactivation (23, 24). The E4A03 trial demonstrated that low-dose dexamethasone (40 mg once weekly) improved survival and reduced serious infections compared with high-dose dexamethasone (40 mg on days 1–4, 9–12, and 17–20) (25), a finding further supported by the MAIA frailty subgroup, where grade 3–4 infection rates reached 41.7% with daratumumab, lenalidomide, and continuous dexamethasone versus 27.7% with lenalidomide and dexamethasone alone (26). Consistently, the IFM2017-03 trial showed that limiting dexamethasone to the first two cycles yielded comparable infection rates (19% versus 21%) despite a markedly longer treatment duration, supporting early dexamethasone discontinuation as a strategy to mitigate infectious morbidity (27).

*Cytotoxic drugs*: Drugs such as cyclophosphamide, vincristine, doxorubicin, and other cytotoxic agents such as cisplatin, etoposide, and melphalan used in protocols such as PACE and conditioning regimens for autologous stem cell transplant (ASCT) have a myelosuppressive effect by disrupting cell division and inducing apoptosis, which can lead to severe and sometimes prolonged neutropenia. The risk of infection also increases with the combined use of corticosteroids (20). Furthermore, treatment with high-dose melphalan prior to ASCT can cause severe mucositis, weaken the mucosal barrier, and create a potential site for bacterial translocation (21).

*Proteasome inhibitors (PIs):* As a drug class, bortezomib, carfilzomib, and ixazomib are associated with hematologic toxicity, including neutropenia and lymphopenia (28). Patients treated with PIs, especially bortezomib, are at higher risk of VZV reactivation, likely due to inhibition of the transcription factor NF-κB, which is essential for T- and B-cell development. This causes a temporary decline in mature CD4+ lymphocytes and promotes T-cell apoptosis, leading to an increased incidence of VZV infections (29).

*Immunomodulatory drugs (IMiDs) and cereblon E3 ligase modulators (CELMoDs):* Thalidomide and its derivatives, such as lenalidomide and pomalidomide, act as anti-MM agents by inhibiting proliferation, disrupting MM cell–stromal interactions, and downregulating key cytokines and growth factors. More recently, CELMoDs have been developed, exerting anti-myeloma effects through enhanced cereblon-mediated degradation of transcription factors and immune activation (30). However, all these drugs have been associated with neutropenia and increased risk of infection. Grade 3–4 neutropenia occurs in approximately 23% of lenalidomide-treated patients and in 32–58% of those receiving pomalidomide (31). Iberdomide and mezigdomide further increase the risk of neutropenia, up to grade 3–4, as well as the risk of bacterial pneumonia, invasive aspergillosis, and CMV reactivation (32, 33). The underlying mechanism remains incompletely understood, although IMiDs have been shown to downregulate PU.1, a transcription factor critical for neutrophil differentiation, thereby impairing granulopoiesis despite preserved myeloid precursors (34).

*Selinexor*: As an oral selective inhibitor of nuclear export that blocks the nuclear export protein exportin 1 (XPO1), selinexor forces nuclear retention and activation of tumor suppressive proteins and prevents the translation of oncoprotein mRNAs. Ultimately, this leads to apoptosis in malignant cells without affecting other cells (35). Neutropenia grade 3 or higher occurs in approximately 20–25% of patients and is generally manageable with G-CSF. Opportunistic infections have been uncommon (36).

*Monoclonal antibodies (mAbs):* Daratumumab and isatuximab, which target CD38, are standard components of both frontline and RRMM regimens, where their addition to dexamethasone, IMiDs, and PI-based combinations has significantly improved prognosis (37). CD38 is a transmembrane glycoprotein highly expressed on malignant plasma cells, as well as on normal plasma cells, activated B, T, and NK cells, and myeloid-derived suppressor cells (MDSCs), making off-tumor immune depletion an inherent consequence of treatment (38, 39). Daratumumab reduces polyclonal IgA, IgE, and IgM by depleting normal plasma cells, whereas IgG levels remain relatively stable due to CD38 downregulation. Ongoing B-cell differentiation supports immunoglobulin recovery and preserves the capacity to mount protective responses to vaccination (39, 40). In addition, daratumumab upregulates the number and activity of cytotoxic T cells and interferon-gamma production, supporting a more effective adaptive immune response (38, 41). A meta-analysis of five phase III RCTs demonstrated that daratumumab combinations significantly increased the risk of neutropenia and leukopenia (all-grade and grade 3–4), with similar findings reported for isatuximab (42, 43). Concomitant use of lenalidomide or pomalidomide may further exacerbate neutropenia, often requiring dose adjustments (16, 44). In a meta-analysis of over 5,000 patients, anti-CD38 mAbs significantly increased the risk of severe infection (HR: 1.27; 95% CI: 1.17–1.37) and severe pneumonia (HR: 1.38; 95% CI: 1.09–1.75); however, despite a cumulative severe infection incidence of 28%, infection-related mortality remained low and comparable to controls (1.59% vs. 1.14%) (41).

Elotuzumab, a signaling lymphocytic activation molecule family member 7 (SLAMF7)-targeting mAbs used with IMiDs in RRMM, selectively activates NK cells by binding to its adapter protein EAT-2, despite SLAMF7 expression on multiple immune cell types (45, 46). Elotuzumab did not increase neutropenia rates but was linked to a higher incidence of grade 3/4 lymphopenia (77% vs. 49% in ELOQUENT-2 and 8% vs. 2% in ELOQUENT-3), possibly indicating changes in lymphocyte trafficking, including NK cells (47, 48). A meta-analysis found a modestly increased risk of infection with elotuzumab, driven predominantly by VZV reactivation (4.1% vs. 2.2% in ELOQUENT-2; 5% vs. 2% in ELOQUENT-3) (49).

*Antibody–Drug Conjugates (ADCs):* Belantamab mafodotin (belamaf) is a monoclonal antibody against the B-cell maturation antigen (BCMA) conjugated to the cytotoxic agent monomethyl auristatin F (MMAF). BCMA is a cell-surface receptor protein that is highly expressed on late-stage B cells and malignant plasma cells, with low levels of expression on hematopoietic stem cells and no expression in non-hematopoietic cells (50). In the DREAMM-7 and DREAMM-8 trials, infections remained among the most frequent adverse events, with pneumonia as the predominant severe infection, and opportunistic infections remained uncommon (51, 52).

*Bispecific antibodies (BsAbs):* In the MM setting, BsAbs target either BCMA or alternative antigens. BCMA-targeting agents include teclistamab and elranatamab, while non-BCMA targets include G-protein coupled receptor family C group 5 member D (GPRC5D), targeted by talquetamab, and Fc Receptor-Like 5 (FcRH5), targeted by cevostamab. GPRC5D has no well-defined function, whereas FcRH5 is involved in B-cell proliferation (8, 53). Treatment with BsAbs carries a considerably higher infectious burden than conventional MM treatment, with a reported cumulative infection risk of 70% (54, 55). A review of 1,143 RRMM patients found all-grade infections in 50% of patients, with 24.5% experiencing grade ≥3 events and an infection-related mortality rate of 25.5% (8). Infections represent a leading cause of non-relapse mortality in this population, underscoring the clinical significance of infectious complications beyond their frequency alone. This high infectious burden with BsAbs reflects both patient- and treatment-related factors. Patients receiving BsAbs are typically heavily pre-treated and already profoundly immunosuppressed, with lymphopenia and hypogammaglobulinemia (53). BsAbs therapy further compounds immune dysfunction through multiple mechanisms: Grade ≥3 neutropenia occurs in approximately one-third of patients, impairing innate defense against bacterial and fungal pathogens; treatment-induced lymphopenia and stimulation of regulatory T cells disrupt adaptive immunity; hypogammaglobulinemia develops in up to 75% of patients, eliminating humoral protection; and persistent antigen stimulation drives T-cell exhaustion, rendering effector T cells dysfunctional over time (56–59). Cytokine release syndrome (CRS), a known adverse effect of BsAbs, causes massive cytokine release, leading to immunoparesis and increased susceptibility to infection. Paradoxically, its treatment usually involves corticosteroids, tocilizumab, or anakinra, which further increases immunosuppression, similar to the management of immune effector cell-associated neurotoxicity syndrome (ICANS) (56–58). Significant reported infections varied across drugs, including PJP with teclistamab, CMV reactivation with elranatamab, and *Helicobacter pylori* gastritis with talquetamab (8, 56–58). Propensity for infections is mainly associated with defects in T-cell and/or B-cell immunity, with an increased risk of PJP and reactivation of CMV and hepatitis B virus (HBV) (56, 57). Compared with non-BCMA-targeted BsAbs, BCMA-targeting agents are associated with significantly higher rates of grade ≥3 neutropenia (39.2% vs. 25.3%; *p* = 0.001) and grade ≥3 infection (30% vs. 11.9%; *p* = 0.01). This disparity reflects the critical role of BCMA signaling in the survival of long-lived plasma cells and subsequent immune reconstitution: Loss of BCMA-dependent plasma cell survival not only depletes immunoglobulin-secreting cells but also impairs the restoration of humoral immunity during and after therapy (8, 60). Cumulative infection rates may be higher with BCMA-targeting BsAbs than with BCMA-directed CAR-T cell therapy (25% vs. 5%; *p* = 0.012), likely reflecting the continuous nature of BsAbs therapy rather than a single exposure, which leads to more profound and prolonged B-cell aplasia and hypogammaglobulinemia (56).

*Chimeric antigen receptor T cells (CAR-T cells):* CAR-T cells are autologous T cells genetically engineered to express a chimeric antigen receptor (CAR), a synthetic construct that recognizes a tumor-associated antigen and triggers T-cell activation via CD3 signaling, independently of major histocompatibility complex presentation. In MM, two different CAR-T cells targeting BCMA, idecabtagene vicleucel (ide-cel) and ciltacabtagene autoleucel (cilta-cel), have been approved for patients who have received ≥3 prior lines of therapy, with cilta-cel additionally approved for lenalidomide-refractory patients following one to three prior lines of therapy based on the phase 3 CARTITUDE-4 trial (61, 62). The elevated risk of infection in this setting is multifactorial. Lymphodepletion chemotherapy induces profound neutropenia and lymphopenia, while BCMA targeting depletes normal antibody-producing plasma cells, leading to severe hypogammaglobulinemia (60, 61, 63). The immunological consequences of immunoglobulin depletion are subclass-specific: IgG2 depletion increases the risk of bacterial infections, whereas low levels of IgG1/IgG3 predispose to viral infections (64). This nuanced pattern of humoral immunodeficiency underscores the importance of monitoring individual immunoglobulin subclasses rather than total IgG alone when assessing infection risk. A key conceptual framework emerging from the CAR-T literature is that infectious risk follows a temporally structured, phase-specific pattern, with distinct pathogen profiles corresponding to the dominant immunological perturbation at each stage (65–67). Pre-infusion events are driven primarily by prior therapy and bridging treatment. In a real-world cohort, 22.5% of patients had experienced an infection in the 30 days preceding CAR-T infusion, highlighting a precarious immune baseline at the time of treatment (66). Patients entering the infusion phase with active or recent infections represent a particularly high-risk subgroup. Early post-infusion (days 0–30) infections are driven primarily by lymphodepletion and neutropenia. In a real-world cohort, it was found that 28 of 60 total infection episodes occurred within the first 30 days after infusion, and bacterial infections were significantly more common in this early period (*p* = 0.02), driven predominantly by gram-negative organisms during neutropenia and mucosal barrier damage, identified in 25% of patients, a higher rate than previously reported (65, 66). Later events (beyond day 30) reflect delayed immune reconstitution. Persistent lymphopenia, T-cell dysfunction, and hypogammaglobulinemia resulting from prolonged B-cell and plasma cell depletion become the dominant immune deficits, driving susceptibility to opportunistic pathogens such as PJP, CMV, and *Aspergillus* spp. (16, 59, 64, 65, 68). The development of CRS, as well as CRS and ICANS treatment, contributes to immunosuppression and consequently infection (64, 69). However, real-world evidence with ide-cel did not support an increased risk of infection with CRS treatment (70). Infection rates in clinical trials of BCMA-directed CAR-T cell therapy in RRMM range from 59% for cilta-cel in CARTITUDE-1 (grade ≥3 in 23%), 62% for cilta-cel in CARTITUDE-4 (grade ≥3 in 26.9%), to 69% for ide-cel in KarMMA (grade ≥3 in 22%) (71, 72). Temporally, bacterial infections predominate in the first 30 days, driven by neutropenia; viral infections emerge after day 30 as lymphopenia and hypogammaglobulinemia prevail; and fungal infections, including PJP, occur sporadically throughout, mainly associated with neutropenia and prolonged corticosteroid therapy (73). A multicenter real-world analysis of 80 RRMM patients treated with either ide-cel (n = 42) or cilta-cel (n = 38) outside of clinical trials reported infections in 71.3% overall, 66.7% with ide-cel, and 76.3% with cilta-cel. The median time to first infection was 38.5 days (range 0–200). The infectious burden carried significant clinical consequences: 33.3% of affected patients required inpatient hospitalization, 5.3% required intensive care unit admission, and infection-related mortality occurred in 7.0% of patients, corresponding to an overall non-relapse mortality of 8.8%, consistent with larger meta-analytic estimates. Among non-relapse deaths, infection represented the leading attributable cause (66, 74, 75).

## Screening for infection

Baseline screening for latent and active infection before treatment initiation remains standard of care and should be repeated at major treatment transitions, particularly before ASCT, lymphodepletion, and T-cell redirecting therapy. Every patient should undergo a comprehensive evaluation, including medical history with risk factors (for example, epidemiological background and past travel history) and vaccination status, as well as a physical examination, before starting treatment. If an infection is detected, a multidisciplinary discussion should be held to weigh the risk of infection worsening with therapy against the potential progression of the underlying malignancy, thereby helping determine the best course of action. Screening assessments, summarized in Table 2, should be updated before each new treatment line and 10–30 days before stem cell collection, transplant, and lymphodepletion. However, some of these tests may be performed earlier and do not need to be repeated.

**Table 2:** Pre-treatment screening for infection in patients with multiple myeloma.

Test/evaluation	Indication/population	Timing/notes
Serological screening		
HBV serology (Anti-HBc, anti-HBs, and HBsAg)	All patients	Before each treatment line. HBsAg^+^: initiate antiviral prophylaxis before immunosuppressive therapy. HBsAg^−^/anti-HBc^+^: monitor HBV-DNA throughout treatment; initiate prophylaxis if reactivation detected. Isolated anti-HBs without documented vaccination: managed as if they were anti-HBc + .
Anti-HCV antibody	All patients	Before each treatment line. If positive, quantify HCV-RNA and start antiviral treatment.
VZV serology (IgG)	Prior to PI, anti-CD38, BsAb, CAR-T, and ASCT	Seronegative patients are candidates for VZV vaccination before immunosuppression.
CMV*-*DNA	Prior to BsAb, CAR-T, or ASCT	Baseline quantification. High-risk CAR-T recipients: weekly surveillance PCR for the first 2–6 weeks post-infusion.
HIV serology	All patients	Before treatment initiation. If positive, refer to infectious consult to start antiretroviral therapy.
Tuberculosis screening		
TST and IGRA	All patients; prioritize those from endemic regions or with prior TB exposure	Before treatment. Positive result: rule out active TB disease with symptom assessment and chest imaging (X-ray or CT) before initiating LTBI prophylaxis.
Immune assessment		
Serum immunoglobulins (IgG, IgA, and IgM)	All patients; mandatory before BsAb or CAR-T	Baseline and monthly monitoring during treatment. IgG < 4 g/L or recurrent infections (e.g., ≥2 pneumonias/year) regardless of level: Ig replacement therapy.
Lymphocyte subsets (CD4, CD8, CD4/CD8 ratio, and NK cells)	Prior to BsAb or CAR-T	Baseline assessment. CD4 count guides PJP prophylaxis duration: continue until CD4 > 200/mm^3^.
Clinical history and physical examination		
Vaccination history	All patients	Identify immunization gaps. Prioritize pre-treatment vaccination when feasible.
Prior infection history (frequency, severity, and pathogens)	All patients	Recurrent or severe infections (e.g., ≥2 pneumonias/year and opportunistic organisms) may indicate underlying immunodeficiency and prompt Ig replacement or prolonged prophylaxis regardless of quantitative IgG level.
Performance status and comorbidities	All patients	Renal impairment (antiviral/antifungal dosing adjustment), respiratory disease (PJP/fungal risk), and diabetes (increased susceptibility to bacterial and fungal infections) should inform prophylaxis selection and intensity.

ASCT, autologous stem cell transplantation; BsAb, bispecific antibody; CAR-T, chimeric antigen receptor T-cell therapy; CMV, cytomegalovirus; HBV, hepatitis B virus; HCV, hepatitis C virus; HIV, human immunodeficiency virus; IGRA, interferon-gamma release assay; Ig, immunoglobulin; LTBI, latent tuberculosis infection; PI, proteasome inhibitor; PJP, Pneumocystis jirovecii pneumonia; TST, tuberculin skin test; VZV, varicella-zoster virus.

## Prophylaxis

The overall prophylactic strategy by treatment class is summarized in Table 3, and the recommended antimicrobial prophylactic agents, doses, and treatment durations are detailed in Table 4.

**Table 3:** Recommended prophylaxis strategy by treatment class in multiple myeloma.

Therapy class	Main immune defect	Major infection risk	Antibacterial prophylaxis	Antiviral prophylaxis	Antifungal prophylaxis	PJP prophylaxis	Ig replacement
Conventional agents							
Conventional chemotherapy *(including pre-ASCT conditioning)*	Neutropenia; mucosal barrier disruption	Bacterial (gram+/gram−); Candida spp.; HSV reactivation	Not routine	Recommended (HSV/VZV)	Selected (ASCT)	Selected	Risk-based
Proteasome inhibitors *(bortezomib, carfilzomib, and ixazomib)*	T-cell dysfunction; transient CD4+ depletion	VZV reactivation; bacterial infections	Not routine	Recommended (HSV/VZV)	Not routine	Selected	Risk-based
IMiDs/CELMoDs *(thalidomide, lenalidomide, and pomalidomide)*	Neutropenia; impaired neutrophil maturation	Bacterial infections; pneumonia	Not routine	Selected	Not routine	Selected	Risk-based
Monoclonal antibodies							
Anti-CD38 mAbs *(daratumumab and isatuximab)*	NK cell & plasma cell depletion; hypogammaglobulinemia	Viral and bacterial; encapsulated bacteria	Selected	Recommended	Not routine	Selected	Consider in recurrent infections
ADCs *(belantamab mafodotin)*	BCMA+ plasma cell depletion; compounded by combination partner	Hypogammaglobulinemia-driven; pneumonia	Selected	Recommended	Not routine	Selected	Consider
T-cell redirecting therapies							
Bispecific antibodies *(BCMA, GPRC5D, and FcRH5-targeting)*	B-cell aplasia; T-cell exhaustion; hypogammaglobulinemia; CRS/ICANS	Opportunistic; viral; bacterial; high infection-related mortality	Selected (early therapy)	Recommended	High-risk	Recommended	Recommended
CAR-T cell therapy *(ide-cel and cilta-cel)*	Lymphodepletion; profound humoral deficiency; CRS/ICANS	Early (D0–30): bacterial → Late (>D30): viral & opportunistic	Selected (during neutropenia)	Recommended	High-risk	Recommended	Recommended

Recommended—indicated for all patients in this treatment category; Selected/Risk-adapted—indicated based on individual risk factors; Consider/Risk-based—individualized decision; Not routine—not recommended as routine prophylaxis. ADC, antibody–drug conjugate; ASCT, autologous stem cell transplantation; BCMA, B-cell maturation antigen; CAR-T, chimeric antigen receptor T-cell; CELMoD, cereblon E3 ligase modulator; CRS, cytokine release syndrome; FcRH5, Fc receptor-like 5; GPRC5D, G-protein coupled receptor family C group 5 member D; HSV, herpes simplex virus; ICANS, immune effector cell-associated neurotoxicity syndrome; IMiD, immunomodulatory drug; Ig, immunoglobulin; mAb, monoclonal antibody; NK, natural killer; PJP, Pneumocystis jirovecii pneumonia; VZV, varicella-zoster virus.

**Table 4:** Antimicrobial prophylaxis in multiple myeloma: agent, dose, setting, and duration.

Pathogen/target	Agent and dose	Setting/indication	Duration
Antibacterial and tuberculosis prophylaxis			
Bacteria	Levofloxacin 500 mg once daily (preferred over ciprofloxacin when pomalidomide is used) Dose per institutional protocol/local resistance patterns	NDMM induction—high-risk patients (ISS II–III, elevated LDH, poor PS, renal impairment) ASCT—all recipients (peri-transplant) CAR-T—ANC < 500/mm^3^ post-infusion BsAb—all recipients at treatment initiation	NDMM: first 3 months of therapy ASCT: until ANC > 500/mm^3^ CAR-T: during neutropenia; avoid during CRS window BsAb: first month; continue during neutropenia
*M. tuberculosis* (LTBI)	Isoniazid (INH) 300 mg OD + pyridoxine 25–50 mg OD Alternative: Rifampicin 600 mg once daily (if INH contraindicated)-based 4-month course Alternative: INH 9000 mg + rifapentine 900 mg once weekly—3 months	All patients with confirmed LTBI (positive TST or IGRA) before starting MM therapy	INH: 6–9 months (preferred: fewer interactions with anti-MM therapies) Rifampicin: 4 months Start before or concurrent with MM therapy; specialist review recommended
Antiviral prophylaxis			
HSV/VZV	Acyclovir 800 mg twice daily or Valacyclovir 500 mg once daily IV: Acyclovir 5 mg/kg q12h if mucositis or unable to take orally	PI, anti-CD38, ADC, BsAb, CAR-T, ASCT IMiD + corticosteroids or combination regimens	Start with MM treatment General: ≥6 weeks after therapy ends Post-ASCT: up to 1 year Post-CAR-T: ≥6 months (start at lymphodepletion)
HBV	Entecavir 0.5 mg once daily or Tenofovir (TDF) 300 mg once daily	HBsAg^+^ or HBV-DNA^+^: prophylaxis mandatory HBsAg^−^/anti-HBc^+^: serial HBV-DNA monitoring; treat if DNA detectable	Throughout MM therapy + 6–12 months (ideally 1 year) after MM treatment completion
Antifungal prophylaxis and PJP			
Candida spp.	Fluconazole 400 mg once daily (preferred) Alternative: Micafungin 50 mg OD if contraindicated, intolerant or severe mucositis	ASCT: profound/prolonged neutropenia + grade 3–4 mucositis	Until engraftment/ANC recovery
Aspergillus spp.	Posaconazole, voriconazole, or isavuconazole (mold-active: preferred) Posaconazole 300 mg once daily (after loading), voriconazole 200 mg twice daily or isavuconazole 200 mg once daily (after loading) Alternative: Micafungin 50 mg IV OD if contraindicated, intolerant or severe mucositis	CAR-T: neutropenia >28 days, >1 tocilizumab dose, anakinra/siltuximab, high-dose steroids >3 days ASCT/BsAb: per individual risk assessment	During neutropenia and high-risk period Reassess at ANC recovery
Pneumocystis jirovecii (PJP)	TMP/SMX 80/400 mg OD or 160/800 mg 3×/week (first-line) Alternatives: Dapsone 50 mg twice daily Atovaquone 750 mg twice daily or 1,500 mg OD (with food) Pentamidine 300 mg inhaled monthly	RRMM + high-dose corticosteroids (≥40 mg dexamethasone/day ≥4 days/week) All BsAb recipients All CAR-T recipients	BsAb: from therapy initiation until CD4 > 200/mm^3^ CAR-T: from day +30 (or when cytopenias resolve) until 6 months or CD4 > 200/mm^3^ Corticosteroids: for duration of high-dose exposure

ANC, absolute neutrophil count; ASCT, autologous stem cell transplantation; BsAb, bispecific antibody; CAR-T, chimeric antigen receptor T-cell; CD4, cluster of differentiation 4; CRS, cytokine release syndrome; HBsAg, hepatitis B surface antigen; anti-HBc, hepatitis B core antibody; HBV, hepatitis B virus; HSV, herpes simplex virus; IGRA, interferon-gamma release assay; IMiD, immunomodulatory drug; INH, isoniazid; ISS, International Staging System; IV, intravenous; LDH, lactate dehydrogenase; LTBI, latent tuberculosis infection; MM, multiple myeloma; NDMM, newly diagnosed multiple myeloma; OD, once daily; PI, proteasome inhibitor; PJP, Pneumocystis jirovecii pneumonia; PS, performance status; RRMM, relapsed/refractory multiple myeloma; TDF, tenofovir disoproxil fumarate; TMP/SMX, trimethoprim/sulfamethoxazole; TST, tuberculin skin test; VZV, varicella-zoster virus.

### Antibiotic prophylaxis

The benefit of routine antibacterial prophylaxis remains uncertain as several clinical trials have shown no significant difference in rates of severe bacterial infection or death between antibiotic prophylaxis and observation alone, with the caveat of an increased risk of growth of multidrug-resistant bacteria (76–78). The Phase 3 trial TEAMM compared levofloxacin prophylaxis (500 mg daily) to placebo for 12 weeks. Although an 8% lower rate was observed with levofloxacin prophylaxis, driven by lower rates of febrile episodes (with 19% episodes in the prophylaxis group vs. 27% in the placebo group), there was no significant difference in deaths due to infection at 12 weeks or in overall survival at 12 months (90% vs. 91%, *p* = 0.94) (79).

A systematic review and meta-analysis evaluating the use of prophylactic antibiotics in patients with MM showed that antibiotic prophylaxis for a finite duration can decrease the overall incidence of infection within the first 3 months following diagnosis (80). Nonetheless, a tiered, risk-adapted approach to antimicrobial prophylaxis is recommended, and consideration of a quinolone should be based on the degree and duration of neutropenia and global risk (81). Apart from the patients’ risk for bacterial infection, the local epidemiology should also be taken into consideration. In communities with a high incidence of fluoroquinolone resistance, the benefits may be outweighed by the risks (tendinitis and tendon rupture, peripheral neuropathy, serious central nervous system effects, and aortic aneurysm and dissection).

In patients at high risk of early infection, namely in patients with high tumor burden (ISS II–III), markedly high concentrations of serum lactic dehydrogenase, poor performance status, and impaired renal function, the use of antimicrobial prophylaxis with fluoroquinolone (e.g., ciprofloxacin 500 mg once daily or 250 mg twice daily) during the first 3 months of therapy can be considered (76, 79, 82). Due to *CYP1A2* inhibition, ciprofloxacin may increase pomalidomide exposure and toxicity. In this case, levofloxacin 500 mg once daily should be preferred (23). In relapsed/refractory MM, the optimal use and duration of antibacterial prophylaxis remain undefined (22). At this stage, there is no consensus regarding the use of antibacterial prophylaxis during this treatment period (64, 83–85).

The role of antibiotic prophylaxis during ASCT until hematopoietic recovery remains debated. While it has been shown to reduce rates of fever and bloodstream infections, this has not translated into a mortality benefit (16, 57, 79). In this context, the decision to adopt prophylaxis should be guided by local antimicrobial resistance profiles, balancing the modest clinical benefit against the risk of selecting for multidrug-resistant organisms.

Regarding more recent immunotherapy, including monoclonal and bispecific antibodies, as well as CAR-T cell therapy, data on infection prevention are scarce, and current practice is largely guided by expert consensus (64). A panel from the Academic Consortium to Overcome Multiple Myeloma through Innovative Trials (COMMIT) recommend antibacterial pharmacological prophylaxis (e.g., levofloxacin 500 mg daily) for recipients of CAR-T cell therapy when absolute neutrophil count (ANC) < 500/mm3 should continue for the period of neutropenia, and in BsAbs at the onset of treatment and continuing for the first month (16, 64, 86, 87). However, the decision-making process should consider local patterns of antimicrobial resistance and the emergence of multidrug-resistant bacteria.

### Tuberculosis infection treatment and prophylaxis

The risk of tuberculosis (TB) in MM patients was found to be 3.11 times higher than in other malignancies, with a 2.03 times increased risk for mortality (88). Before initiating preventive therapy, active TB disease must be ruled out by evaluating clinical symptoms and performing lung imaging. Given the inherent immunosuppression in MM, diagnosis relies on a combination of the tuberculin skin test and an interferon-gamma release assay (IGRA) to maximize sensitivity. TB infection is defined as a positive result in either test without evidence of active disease. The preferred prophylactic regimen is isoniazid 300 mg daily for 6–9 months, combined with pyridoxine supplementation due to the cumulative risk of neuropathy in MM patients. Rifampicin-based alternatives are generally avoided due to significant interactions with PIs, IMiDs, and corticosteroids commonly used in MM therapy (89). Patients should be reevaluated during MM treatment in case of contact with an active TB case, and prophylaxis should be considered in high-risk contacts.

### Antiviral prophylaxis

The use of PIs and anti-CD38 mAbs is associated with high rates of viral reactivation, particularly among the *Herpesviridae* family (herpes simplex virus and VZV) (13, 90, 91). The role of IMiDs in viral reactivation is less well understood than that of PIs and anti-CD38 mAbs, especially in monotherapy (92). However, there are reported cases of VZV and hepatitis B virus (HBV) reactivation with the use of second and third generation IMiDs, some of them with fatal outcomes (93, 94). Patients undergoing intensive chemotherapy (e.g., PACE) and high-dose chemotherapy followed by ASCT are also at increased risk of viral reactivation. Recent therapies targeting the B-cell maturation antigen, such as BsAbs, ADCs, and CAR-T, are also associated with an increased risk of viral reactivation, reflecting profound humoral deficiency and delayed immune reconstitution (16, 57). Therefore, antiviral prophylaxis against VZV virus reactivation is recommended in MM patients undergoing therapy with PIs, anti-CD38 mAbs, ADCs, BsAbs, CAR-T cells, and in patients ASCT. In patients undergoing monotherapy with IMiDs, other risk factors, such as frailty, tumor burden, and the cumulative immunosuppressive effect of previous lines of treatment, should also be considered. If the IMiD is associated with any of the above drugs or corticosteroid therapy, antiviral prophylaxis is recommended. Antiviral prophylaxis is advised for all patients with recent VZV history or severe/recurrent HSV infection, regardless of MM type therapy. Prophylaxis should be carried out with acyclovir at a dose of 800 mg twice daily, or valacyclovir 500 mg daily, and should be started with MM treatment. During ASCT and in the setting of mucositis, or in the absence of oral intake, acyclovir 5 mg/kg q12h is recommended. It is suggested that prophylaxis be maintained for at least 6 weeks after the end of therapy and for up to 1 year after ASCT if no further maintenance is required (16, 23, 57). In a patient undergoing CAR-T, VZV prophylaxis should start during lymphodepletion therapy and continue for 6 months post-CAR-T (87, 95). This period may be extended depending on risk factors such as disease response, therapeutic lines used, and their intensity (16, 96). Monitoring for HSV/VZV reactivation is generally not recommended, while prophylactic treatments are in use (57).

Hepatitis B reactivation may occur in patients with MM depending on HBV serological status. HBV reactivation, if it occurs, may be associated with high morbidity and mortality (97, 98). Therefore, all patients proposed for therapy should undergo serological testing for hepatitis B, including hepatitis B surface antigen (HBsAg) and hepatitis B core and surface antibodies (HBcAb and HBsAb, respectively) (13, 99). In patients with positive HBsAg or with negative HBsAg and positive anti-HBc, the level of circulating HBV deoxyribonucleic acid (DNA) should be quantified. In patients with chronic hepatitis B infection (HBsAg positive) or occult hepatitis B infection (HBsAg negative and HBV-DNA quantification is positive), antiviral therapy is recommended. In patients with resolved hepatitis B infection or functional cure (HBsAg negative, HBcAb positive, and HBV-DNA quantification undetectable), prophylaxis should be prescribed in the following situations: hematopoietic stem cell transplant, CAR-T cells, bispecific antibodies, CD38 monoclonal antibodies, high-dose combination chemotherapy, B-cell depleting therapies, anthracyclines, and high-dose corticotherapy (superior to 40 mg of prednisolone per day). In patients with functional cure of hepatitis B, when prophylaxis is not prescribed, every 3 months, HbsAg and HBV-DNA quantification should be performed throughout treatment to monitor disease progression. Caution is advised in patients without an HBV vaccination registry who have isolated HBsAb positivity; there are reports of HBV reactivation with immunosuppression as this may represent a resolved hepatitis B infection with loss of HBcAb (100). In HBV treatment or prophylaxis, high-barrier-to-resistance nucleos(t)ide analogs, such as entecavir (0.5 mg daily), tenofovir disoproxil fumarate (300 mg daily), or tenofovir alafenamide (25 mg daily), are recommended as they are effective, have a low resistance rate, and are well tolerated (101). Antiviral prophylaxis should be initiated before or concomitantly with immunosuppressive treatment and continued throughout therapy and for 12–18 months, or preferably until 1 year after withdrawal of MM treatment (23, 57, 87, 95).

As recommended for hepatitis B serological screening, all patients about to start therapy should undergo hepatitis C serological screening. Exacerbation of chronic Hepatitis C has been documented in the context of MM treatment (102). HCV-positive patients should proceed with the study of circulating HCV-RNA quantification to document chronic hepatitis C. If a chronic infection is reported, it is advisable to initiate antiviral therapy, in accordance with international recommendations, preferably before the onset of MM-directed treatment (102, 103). Patients undergoing prophylaxis or active treatment for HBV or HCV should be referred to and monitored by a specialist in infectious diseases and/or hepatology.

In patients undergoing various lines of therapy, including BsAbs, and in patients undergoing ASCT, cytomegalovirus (CMV) reactivation has been documented. Routine prophylaxis for CMV reactivation is not recommended for most patients undergoing active treatment for MM. Surveillance is reasonable in selected high-risk populations, particularly after CAR-T cell therapy and during BCMA-directed BsAb treatment. However, if the risk of CMV infection is suspected, baseline quantification and monthly CMV PCR to monitor CMV replication are recommended (16, 57). In patients undergoing CAR-T cell therapy, weekly CMV surveillance should be considered during the first 2–6 weeks post-infusion in those at high risk of reactivation. High-risk criteria include a history of ASCT or prior BCMA-targeted therapy, or the need for corticosteroids for more than 3 days post-CAR T-cell infusion for CRS/ICANS (87, 95). Letermovir remains investigational in this setting and cannot yet be recommended routinely outside selected institutional practice. Treatment of CMV reactivation is recommended with oral valganciclovir, intravenous ganciclovir, or foscarnet (57).

Prophylaxis for Epstein–Barr virus (EBV) and human herpesvirus 6 (HHV-6) is also not recommended (9).

### Antifungal prophylaxis

Although invasive aspergillosis was prevalent during the era of conventional chemotherapy, its incidence fell below 1% with the introduction of novel agents (104, 105). However, a retrospective study of 372 patients reported an overall invasive fungal infection rate of 2.4%, rising markedly to 15% in those who had received three or more prior lines, and emerging data suggest a further upward trend with cellular therapies (65, 68). As the epidemiological landscape continues to evolve, the range of antifungal approaches has grown. Antifungal prophylaxis practices vary considerably across institutions, driven by differences in local fungal epidemiology and endemic flora. These institutional factors should always be considered when selecting an antifungal prophylaxis strategy. In fact, new challenges have arisen, including increasing resistance in Candida and Aspergillus species and a changing pattern of non-Aspergillus mold infections (106).

Anti-yeast (e.g.*, Candida* spp.) prophylaxis with an oral triazole or parenteral echinocandin is recommended for patients at risk for profound and prolonged neutropenia and grade 3/4 mucositis, such as those undergoing ASCT, in whom the risk of invasive candidiasis is high (86). Azoles are the primary medications used to prevent fungal infections. Fluconazole at a dose of 400 mg daily is popular due to its affordability and low toxicity. Studies have demonstrated that fluconazole prophylaxis effectively decreases fungal colonization, invasive fungal infections, and related mortality in ASCT patients (107). Micafungin 50 mg daily is recommended for azole-intolerant patients undergoing ASCT due to oral mucositis (16, 23).

Antimold therapy (e.g., *Aspergillus* spp.) may be considered for selected high-risk patients, particularly when the risk of invasive aspergillosis exceeds 6%. Fluconazole 400 mg daily, the most used drug in antifungal prophylaxis, is inactive against most molds. Examples of mold-active agents include echinocandins and other azole antifungals, such as posaconazole, voriconazole, and isavuconazole (86). Routine fungal testing (such as *β*-glucan or galactomannan tests) is not usually recommended due to the low diagnostic yield in patients receiving prophylaxis or without a high risk of fungal infection. However, if aspergillosis is suspected, serum galactomannan testing is advised (57).

In the context of CAR-T cell therapy, it is reasonable to consider fluconazole 400 mg daily prophylaxis for high-risk patients. This includes patients with prolonged and severe neutropenia lasting beyond 28 days until neutrophils recover, those who have received more than one dose of tocilizumab, and those receiving second-line agents such as anakinra or siltuximab. Additionally, high-dose or extended steroid (more than 3 days of dexamethasone at over 10 mg or methylprednisolone at 1 g daily) to manage CRS or ICANS also supports prophylaxis. Micafungin 50 mg daily can be considered as an alternative to fluconazole if it is contraindicated (64, 87, 95, 108).

PJP is a life-threatening opportunistic fungal infection that can affect patients with MM. Despite its rarity in this setting, it is associated with a mortality rate of 30–60%, mainly after ASCT (16, 109). Anti-Pneumocystis prophylaxis is indicated when the risk for PJP is >3.5% (86). Consensus guidelines and recommendations for infection prevention in MM by the IMWG and the European Conference on Infections in Leukemia (ECIL) recommend PJP prophylaxis with trimethoprim/sulfamethoxazole (TMP/SMX) in patients with RRMM and for those who received high dosages of corticosteroids (e.g., ≥40 mg of dexamethasone by day for ≥4 days per week). Anti-PJP prophylaxis is universally recommended for all patients undergoing BsAbs or CAR-T cell therapy due to the high mortality and observed prevalence in clinical trials (57). The recommended treatment schedule for TMP-SMX is trimethoprim 80 mg/sulfamethoxazole 400 mg daily, or trimethoprim 160 mg/sulfamethoxazole 800 mg three times a week. For patients receiving CAR-T cell therapy, treatment should begin 30 days after infusion, or once cytopenias resolve, whichever is later, and should continue for 6 months or until CD4 levels are above 200/mm^3^, whichever is longer. For those receiving BsAbs, PJP prophylaxis is recommended to start at therapy initiation and continue for the duration of treatment or until the CD4 count exceeds 200/mm^3^, whichever is longer (16, 64). Patients with sulfonamide allergies should be given alternatives, such as atovaquone (1,500 mg daily), dapsone (50 mg twice daily), or aerosolized pentamidine (300 mg once monthly). Alternative options to TMP-SMX could also be considered in patients treated with IMiDs due to the potential increased risk of severe skin toxicity. Dapsone should not be given to patients with glucose-6-phosphate dehydrogenase deficiency due to the risk of hemolytic anemia and methemoglobinemia (16, 84).

## Polyclonal immunoglobulin (Ig) replacement (IgRT)

The use of polyclonal natural human immunoglobulin to overcome immunoparesis, which is associated with decreased serum levels of polyclonal IgG and increased susceptibility to infections, is expanding as studies have shown its benefits (110–113). The immune boost triggered by the therapeutic use of low-dose Ig is associated with a direct increase in serum IgG levels and with the expansion of lymphocyte repertoire diversity, which positively impacts lymphocyte function, including their regulation. Several other roles are attributed to these natural antibodies, including defense against infections, clearance of aging cells, T-cell antigen presentation, anti-tumoral surveillance, anti-inflammatory activity, and selection of immune repertoires and the homeostasis of auto-reactivity (114, 115).

Historically, the routine use of IgRT for MM patients, administered intravenously or subcutaneously, was not universally recommended due to conflicting evidence from older studies. However, the landscape has changed significantly with the advent of newer, highly immunosuppressive therapies, such as BCMA-directed bispecific antibodies and CAR-T cells (16, 23, 57). In fact, it was shown that daratumumab treatment can double the incidence of hypogammaglobulinemia (e.g., from 30.3% pre-treatment to 61.4% post-treatment) and that BsAbs, particularly BCMA-targeting BsAbs, are strongly associated with profound and prolonged hypogammaglobulinemia, sometimes approaching an agammaglobulinemic state in responders. Hypogammaglobulinemia incidence in BsAbs trials has been reported as high as 74.5% (teclistamab) to 87% (talquetamab) (7, 111, 113, 116, 117).

A report from the IMWG on recommendations for infection prevention in MM lists Ig as a possible therapeutic option to address immune suppression in these patients (16). A retrospective case-crossover study found that IVIG use was associated with a 39% reduction in all infections and a 72% reduction in serious (grade 3–4) infections in daratumumab-treated patients (111). For patients receiving BsAbs therapy, there is strong evidence supporting the efficacy of IgRT. One study showed a 90% lower rate of grade 3–5 infections during periods when patients received IgRT compared with periods without IgRT (117). Another study on teclistamab reported an 11.6-fold lower incidence of serious infections (0.12 vs. 1.36 per patient-year) and a significantly lower cumulative incidence at 6 months (5.3% vs. 54.8%) in patients receiving IgRT as primary prophylaxis compared to the observation group (118). Another study also observed that IgRT supplementation was associated with improved PFS (median PFS 15 vs. 8 months; *p* = 0.026) and OS (median OS 44 vs. 16 months; *p* = 0.007) (119).

Indications include IgG levels below 400 mg/dL, patients who have experienced two or more severe recurrent infections by encapsulated bacteria (or other pathogens reasonably thought to be due to IgG), regardless of IgG level, and patients with life-threatening infections or documented bacterial infections with no or insufficient response to antibiotic therapy (13, 16, 57). Regular monitoring of immunoglobulin levels (IgG, IgA, and IgM) is crucial, preferably monthly during treatment. However, the frequency of infections should also be monitored as serum levels alone may not fully reflect the capacity to mount an antibody response (57). Recommended doses typically range from 200 to 400 mg/kg every 3–4 weeks, administered intravenously or subcutaneously (13, 16, 120). This dose should be adjusted to maintain IgG levels above 400 mg/dL and keep the patient free of infection (118). More recently, data suggest starting Ig within 1–2 cycles of BsAbs initiation regardless of the IgG levels or infection history. Ig treatment should continue during BsAbs therapy in all patients and for at least 6 months after the last administration in those who have stopped treatment, mainly anti-BCMA BsAbs (64, 113, 121). In these patients treated with BsAbs, Ig should be administered outside the CRS risk window, preferably initiated after completion of step-up dosing as Ig can be associated with signs and symptoms similar to CRS (64, 118). For CAR-T cell therapy recipients, Ig replacement is recommended to start approximately 30 days post-treatment and continue until either 1 year has passed or the serum IgG level exceeds 400 mg/dL, whichever occurs later (64, 87, 95). Live-attenuated vaccines should generally be avoided in immunosuppressed patients. Despite this, vaccine antigens are neutralized by administered immunoglobulins, a phenomenon observed with live vaccines but still mostly theoretical for inactivated vaccines (16, 122–124).

## Use of myeloid growth factors

Treatment-related toxicities may compromise outcomes and lead to dose reductions, delays, or premature discontinuation, with neutropenia being a hematologic side effect commonly associated with chemotherapy (125, 126). Granulocyte colony-stimulating factor (G-CSF) is generally recommended in afebrile patients when the anticipated risk of fever and neutropenia exceeds 20% (81). This includes individuals undergoing ASCT, CAR-T cell therapy, and conventional chemotherapy (127, 128). G-CSF prophylaxis is recommended for patients on low- or low-intermediate-dose regimens with severe neutropenia. It helps prevent treatment delays, for example, by dosing at short intervals to counteract neutropenia induced by agents such as lenalidomide and pomalidomide (129–131). In most cases, G-CSF at 5 μg/kg is given once daily until the ANC has recovered to at least 1,000 cells/mL on 2 consecutive days, usually with a daily dose of 300 μg or 480 μg for patients weighing over 90 kg or those who do not respond adequately to the standard dose. For patients undergoing autologous bone marrow transplantation, the initial dose of G-CSF is 5 μg/kg daily, starting 5–7 days after high-dose therapy and tapered based on neutrophil counts during post-nadir neutrophil recovery (132). In the context of BsAbs and CAR-T, G-CSF should be avoided during the CRS risk window, as it can trigger cytokine release, and its associated symptoms may be difficult to distinguish from those of CRS. Beyond this window, however, treatment-related neutropenia occurs relatively frequently and can generally be managed safely with G-CSF support (133–135).

## Vaccination

Vaccination is one of the most effective prophylactic measures for protecting against disease, estimated to prevent approximately 2–3 million deaths per year (16, 136). In patients with MM, the response to vaccination is less pronounced than in the general population, especially in those with uncontrolled disease, due to a weakened immune system that affects both cellular and humoral immunity, as well as targeted therapy (137, 138). Despite its lower efficacy, the benefit of vaccination in patients with MM is well established and should be implemented, with measures to optimize the response and to address any contraindications (137, 139, 140). The summary of vaccination options and recommendations in MM is detailed in Table 5.

**Table 5:** Vaccination recommendations in multiple myeloma.

Vaccine	Type	Schedule	Timing considerations	Post-ASCT	Post-CAR-T
Influenza	Inactivated (tetravalent)	Annual; 1 dose (consider 2nd dose 30 days after first to improve seroconversion). High-dose formulation preferred in patients ≥65 years	Vaccinate before seasonal onset; preferred ≥2 weeks before therapy or ≥3–6 months after last cycle	Annual; before seasonal onset post-engraftment	3 months post-CAR-T; vaccinate before CAR-T if feasible (pre-treatment immunity persists)
Pneumococcal	Conjugate + polysaccharide (inactivated)	PCV20, then PPSV23 ≥ 8 weeks later. Repeat PPSV23 every 5 years (≥65 years). If prior PPSV23: give PCV20 ≥ 1 year later	Before therapy or ≥3–6 months after last cycle; avoid during anti-CD38 or BCMA-targeting agents if possible	3 doses PCV20 starting at 3–6 months post-ASCT, then 1 dose PPSV23	≥6 months post-CAR-T (inactivated vaccines)
*Haemophilus influenzae* B (Hib)	Conjugate (inactivated)	1 dose; particularly recommended in patients with functional/anatomical asplenia (amyloidosis, liver involvement)	Before therapy initiation	3 doses, separated by ≥ 4 weeks (initiated 3–6 months post-ASCT)	3 doses, separated by ≥ 4 weeks (initiated 6 months post-CART)
Meningococcal	Conjugate (A, C, Y, W-135) + recombinant (serogroup B)	Tetravalent MenACWY conjugate vaccine + MenB protein-based vaccine; 2 doses, 8 weeks apart	Before therapy; MM confers HR 16.6 for invasive meningococcal disease vs. general population	Recommended post-engraftment	≥6 months post-CAR-T
COVID-19 (SARS-CoV-2)	mRNA (bivalent preferred)	Primary series (3 doses) + booster ≥4 months after 3rd dose; update with adapted bivalent vaccines as new variants emerge	Response suboptimal with anti-CD38, BCMA-targeting agents; vaccinate early in disease course if possible	Annual/per current guidance; start ~3 months post-ASCT for seasonal doses	3 months post-CAR-T. Pre-CAR-T vaccination recommended (humoral immunity persists post-infusion); revaccinate 3–4 months post-CAR-T (3 doses every 2 months)
Varicella-zoster virus (VZV)	Recombinant (non-live)—preferred	2 doses, 2–6 months apart. Seroconversion: 81% after dose 1, 90% after dose 2. Revaccinate if previously received live zoster vaccine (≥8 weeks apart)	Preclinical stages (MGUS/SMM) or sustained remission preferred; antiviral prophylaxis should continue alongside vaccination if patients continue treatment	Dose 1 at 50–70 days post-ASCT; dose 2 at 1–2 months later	≥12 months post-CAR-T (when no longer severely immunocompromised)
RSV	Recombinant prefusion F-protein (non-live)	1 dose (per current product labeling); may be co-administered with influenza vaccine	Prioritize before or early in treatment course; particularly in patients ≥60 years. Risk is 16-fold higher in MM vs. general population	Recommend post-engraftment; timing per institutional protocol	Data in CAR-T recipients lacking; consider based on risk–benefit when immune reconstitution achieved

Live vaccines (e.g., yellow fever, MMR, and live-attenuated influenza): Generally contraindicated in MM. Consider only in preclinical stages (MGUS/SMM), >24 months post-ASCT without ongoing immunosuppression, or clear evidence of immune recovery. Avoid within 1 week before or after Ig administration. ASCT, autologous stem cell transplantation; BsAb, bispecific antibody; CAR-T, chimeric antigen receptor T-cell; Hib, *Haemophilus influenzae* type B; MGUS, monoclonal gammopathy of undetermined significance; MMR, measles-mumps-rubella; mRNA, messenger RNA; PCV, pneumococcal conjugate vaccine; PPSV23, 23-valent pneumococcal polysaccharide vaccine; RSV, respiratory syncytial virus; SMM, smoldering multiple myeloma; VZV, varicella-zoster virus.

Given that the immune response to vaccination is compromised in patients with MM, particularly those undergoing active treatment, it is recommended that, whenever possible, patients be vaccinated in the preclinical stages of the disease (monoclonal gammopathy of undetermined significance and smoldering MM) (139, 140). In patients with MM, vaccination should, whenever possible, be initiated 2 weeks before the start of therapy, 3–6 months after the last cycle, or after autologous hematopoietic progenitor cell transplantation (141). The type of therapy at the time of vaccination significantly influences serological response. IMiDs used as monotherapy are associated with better serological responses, whereas the use of corticosteroids, anti-CD38 mAbs, and BCMA is associated with reduced vaccination response (137, 142–146).

Vaccination should generally be delayed until recovery of lymphopenia, usually 3– 6 months, after some treatments, such as CAR-T cells (87, 95, 147). If a patient has achieved remission after CAR-T cell therapy, inactivated vaccines may be considered 6 months later. Live and non-live adjuvant vaccines can be cautiously considered 1 year after CAR-T cell therapy, based on the pattern of immune reconstitution and the patient is no longer being classified as severely immunocompromised. For seasonal infections (e.g., influenza and SARS-CoV-2), vaccinations could be initiated approximately 3 months after CAR-T cell therapy. Importantly, post-CAR-T vaccination is one of the most variable practices in CAR-T treatment and is often based on ASCT protocols (87, 95, 108).

BCMA-directed CAR-T cell therapies cause profound and prolonged plasma cell depletion, directly eliminating the long-lived plasma cells that maintain antibody memory. Recent prospective data demonstrate that up to half of BCMA-targeted CAR-T recipients lose seroprotective titers against common vaccine-preventable pathogens (including *S. pneumoniae*, *H. influenzae* b, and pertussis) within 1 year post-therapy, even when protective antibodies were documented immediately prior to infusion (148, 149). Consequently, current consensus guidelines from the EBMT/JACIE/EHA strongly recommend a complete revaccination series for all patients following CAR-T cell therapy, irrespective of prior transplant or vaccination history (150).

For patients receiving BsAbs therapy, vaccine responses may be more favorable during periods of lower treatment intensity, with longer dose spacing (e.g., every 4 weeks or longer), or during a brief planned treatment pause (124). Indefinite deferral of vaccination on the grounds of ongoing Ig supplementation is not recommended: Passive Ig replacement does not confer active immunity or T-cell memory, may provide a false sense of protection, and cannot compensate for the progressive decline in T-cell responsiveness observed with prolonged BsAbs exposure (123).

In patients with MM, live vaccines are generally contraindicated due to the risk of severe post-vaccination disease. They should only be considered in exceptional cases, for example, in patients at preclinical stages, with sustained disease control, or with clear evidence of immune recovery (e.g., >24 months post-ASCT, without graft-versus-host disease or ongoing immunosuppression). For safety reasons, inactivated and recombinant vaccines are the preferred options (6, 11).

Routine monitoring of vaccine responses is not uniformly recommended, given the high costs, limited availability, technical variability, and lack of standardized methods (6, 48).

*Influenza* vaccine: About half of infectious complications in patients with MM are caused by viral agents, with influenza the most common (151). The most vulnerable populations and those most likely to develop severe infection or death are elderly patients with significant comorbidities or immunocompromised patients, characteristics common in patients with MM. In this context, annual influenza vaccination is highly recommended for all MM patients, their non-immune family members, close contacts, and healthcare workers (13, 16). Tetravalent, inactivated vaccines that protect against strains A and B are recommended. Although there is no consensus on guidelines, studies show that administering a second dose 30 days after the first dose increases seroconversion (137, 152, 153). Tetravalent, inactivated influenza vaccines containing higher antigen concentrations have been used in patients with MM, particularly those aged 65 years or older, to enhance their efficacy (127).

*Pneumococcal* vaccine: The risk of *Streptococcus pneumoniae* infection is increased in cases of decreased IgG production, impaired bacterial clearance, and advanced age, conditions common in patients with MM. Two types of pneumococcal vaccines are available that protect against the most commonly identified serotypes. The typical schedule involves a conjugated vaccine (PCV20) followed by a polysaccharide vaccine (PPSV23) after a specified interval (6–12 months, at least 8 weeks). PPSV23 may be repeated every 5 years for patients aged 65 or older. Patients previously vaccinated with PPSV23 should receive PCV20 1 year later (16, 122).

*Haemophilus influenzae* type B (Hib) vaccine: The risk of severe invasive *Haemophilus influenzae* type b infection is significantly increased in patients with MM. Consequently, a single dose of the Hib vaccine is recommended for patients about to start conventional therapy, particularly those with anatomical or functional asplenia, which can be observed, for example, in cases of amyloidosis or MM with liver involvement (110). Conversely, for patients undergoing intensive cellular therapies, such as ASCT or CAR-T cell therapy, a complete 3-dose series of the Hib vaccine is required. These doses should be separated by at least 4 weeks and are typically initiated 3–6 months post-procedure to maximize immunogenicity during immune reconstitution (122).

Meningococcal vaccine: The diagnosis of MM is among the risk factors identified for invasive meningococcal disease (HR: 16.6 for patients with MM when compared to the general population). Tetravalent capsular polysaccharide conjugate vaccines (A, C, Y, W-135) and protein-based recombinant vaccines targeting serogroup B are available. Vaccination against meningococcal infection appears to be a reasonable option for all patients with MM who are about to start treatment (96, 122).

COVID-19 (SARS-CoV-2) vaccine: Patients with MM have an approximately 2-fold higher risk of SARS-CoV-2 infection than the general population (10) and a higher risk of severe disease, prolonged course, and death. The risk of developing severe infection is higher in patients with uncontrolled disease and in those who have been on therapy for a shorter period (< 3 months) (14). In this context, vaccination against SARS-CoV-2 is recommended for all patients diagnosed with MM (13, 16). However, studies show that the response to vaccination in this population is suboptimal, reinforcing the importance of vaccination in the early stages of the disease (154). Because humoral responses remain attenuated and variant-adapted vaccine formulations continue to evolve, SARS-CoV-2 vaccination in MM should follow contemporary booster-based strategies rather than a fixed historical number of doses. Bivalent vaccines should preferably be used. With the emergence of new variants, adapted vaccines may be developed, and a booster dose is recommended (13, 16, 122).

Seroconversion rates after vaccination are low among CAR-T recipients, ranging from 11 to 29% across studies (155). A prospective multicenter cohort of 65 CAR-T recipients found that SARS-CoV-2 vaccination within 4 months of infusion produces higher antibody responses than vaccination at 4–12 months, while T-cell responses remain similar across timeframes (156). Pre-CAR-T humoral immunity appears to persist after infusion, with previously vaccinated patients exhibiting improved immunoglobulin and T-cell responses following revaccination. This supports the current approach of vaccinating against SARS-CoV-2 before CAR-T and then revaccinating (3 doses every 2 months) starting at 3–4 months post-infusion. Similar evidence backs vaccination against seasonal influenza both before and after CAR-T treatment (157).

Varicella-zoster virus vaccine: The VZV is a DNA virus that remains dormant in dorsal root or trigeminal ganglia after infection. Immunosuppression from the disease and its treatment causes high rates of reactivation (4). Therefore, to reduce the risk of infection and morbidity associated with postherpetic neuralgia, vaccination against VZV is recommended for all patients diagnosed with MM (13, 16). Vaccination should be carried out with a recombinant vaccine (non-live) for safety and efficacy, providing greater and more prolonged protection against VZV. Seroconversion rates of 81% after the first dose and 90% after the second dose have been documented for the recombinant vaccine (158). Two doses of this vaccine are recommended, administered approximately 2–6 months apart. For ASCT recipients, the first dose is typically given 50–70 days after ASCT, and the second 1–2 months later. Revaccination with the recombinant vaccine is recommended for those who previously received the live zoster vaccine, at least 8 weeks apart (16, 57). Due to the risk of developing active disease after vaccination in immunocompromised patients, live vaccines should be considered only in exceptional situations, provided that the patient is in the preclinical stages, such as MGUS and smoldering MM, or has sustained a disease response. Antiviral drug prophylaxis should be maintained in patients vaccinated for VZV because the level of protection from vaccination is difficult to assess, complementing vaccination to reduce the risk of reactivation (16, 57).

Respiratory syncytial virus (RSV) vaccine: RSV is among the high-risk vaccine-preventable infections in MM, with a population-based Swedish registry study of 8,672 patients reporting a 16-fold increased risk compared to healthy controls (159). Although prospective efficacy data in MM patients are not yet available, the magnitude of RSV-associated morbidity, combined with the known clinical effectiveness of other inactivated vaccines in MM, supports a recommendation to offer RSV vaccination to all patients, prioritizing those not yet on active treatment, those in sustained remission, and older patients, who bear the greatest infectious burden (159, 160). The RSV vaccine can be administered concurrently with the influenza vaccine (161). Prospective data on RSV vaccine immunogenicity and clinical efficacy in MM patients, especially those receiving new immunotherapies such as BsAbs and CAR-T cells, are currently lacking and urgently needed to inform clear recommendations.

## Conclusion

Infection risk in MM is dynamic, cumulative, and strongly treatment-specific. As the shift from traditional cytotoxic regimens to T-cell redirecting therapies continues, the infectious landscape has changed: Bacterial pneumonia and herpes reactivation are now complemented by risks such as profound B-cell aplasia, persistent hypogammaglobulinemia, and late opportunistic infections in patients on BsAbs and CAR-T therapies. Understanding this shift and therapy-specific infection phenotypes is essential for developing effective prevention strategies. Risk-adapted approaches integrating disease stage, immune competence, and treatment modality can help guide the appropriate use of antimicrobial prophylaxis, vaccination, and immunoglobulin replacement. Emerging evidence supports the growing role of immunoglobulin replacement therapy for patients receiving BCMA-targeting therapies and other immunotherapies associated with severe and persistent humoral immune deficiency. Current guidelines highlight that no single prophylactic approach suits all MM patients, and significant gaps persist. There is limited head-to-head comparison of prophylactic strategies for BsAbs and CAR-T, the ideal duration of post-CAR-T antiviral protection remains uncertain, and IgRT dosing needs validation and structured protocols. Vaccination in severely immunosuppressed patients remains challenging, but recent data support continued implementation with individualized timing rather than deferral. Future research should aim to refine immune-based risk stratification tools, optimize prophylactic strategies, and more clearly define the role of immunologic biomarkers in predicting susceptibility to infection. The proposed framework, combining risk stratification, pre-treatment screening, tailored prophylaxis, and reassessment at each therapy change, provides clinicians with a practical, evidence-based strategy to reduce infections, a leading cause of non-relapse mortality in MM, particularly in the era of bispecific antibodies and CAR-T cell therapy.
