# Supplementary material for: Practical recommendations for infectious prophylaxis and vaccination in multiple myeloma patients
Source: Front Med (Lausanne). 2026 Jun 30;13:1847793. doi: 10.3389/fmed.2026.1847793 (PMC13366191; doi:10.3389/fmed.2026.1847793)
Supplement: Supplementary file 1 [file Table_1.DOCX]

# Supplementary Table 1. Reference Support Table for Figure 1

| Panel | Treatment | Drug class | Estimate | Key references |
| --- | --- | --- | --- | --- |
| A | Corticosteroids (high-dose pulses) | Conventional agent | 30% | Refs 22, 23, 24 |
| A | Cytotoxic drugs (incl. ASCT) | Conventional agent | 35% | Refs 20, 21, 22, 23 |
| A | Proteasome inhibitors | Conventional agent | 22% | Refs 22, 23, 28 |
| A | IMiDs / CELMoDs | Conventional agent | 28% | Refs 22, 23, 31, 32, 34 |
| A | Anti-CD38 mAbs | Monoclonal Ab | 28% (grade ≥3) | Ref 23, 31, 41, 42, 44 |
| A | Belantamab mafodotin | Monoclonal Ab | 30% | Refs 6, 51,52 |
| A | BsAbs (all-grade) | T-cell redirecting | 50% (all-grade) | Refs 5, 6, 54, 57, 58 |
| A | BCMA-directed BsAbs (grade ≥3) | T-cell redirecting | 30% (grade ≥3) | Ref 5, 6, 54, 56, 57 |
| A | Non-BCMA BsAbs (grade ≥3) | T-cell redirecting | 12% (grade ≥3) | Ref 5, 54, 56, 60 |
| A | CAR-T (ide-cel / cilta-cel) | T-cell redirecting | 64% (all-grade range) | Refs 6, 57, 61, 67, 68, 72, 73, 74 |
| A | CAR-T (grade ≥3) | T-cell redirecting | 22% (grade ≥3) | Refs 6, 67, 68, 72, 73, 74 |
| B | BsAbs (overall) | T-cell redirecting | Bacterial 55%, Viral 40%, Fungal 5% | Ref 56, 57 |
| B | CAR-T (D0–30) | T-cell redirecting | Bacterial 65%, Viral 25%, Fungal 10% | Refs 67, 68, 74 |
| B | CAR-T (>D30) | T-cell redirecting | Bacterial 30%, Viral 65%, Fungal 5% | Refs 67, 68, 74 |

Reference numbers correspond to the manuscript bibliography. BsAbs = bispecific antibodies; CAR-T = chimeric antigen receptor T-cell therapy; D = day; IMiDs = immunomodulatory drugs; CELMoDs = cereblon E3 ligase modulators; ASCT = autologous stem cell transplant; BCMA = B-cell maturation antigen.
